# Supplementary material for: Excitatory–inhibitory balance within EEG microstates and resting-state fMRI networks: assessed via simultaneous trimodal PET–MR–EEG imaging
Source: Transl Psychiatry. 2021 Jan 18;11:60. doi: 10.1038/s41398-020-01160-2 (PMC7813876; doi:10.1038/s41398-020-01160-2)
Supplement: Supplementary file 1 — Supplementary Table 1 [file 41398_2020_1160_MOESM1_ESM.docx]

**Supplementary Data**

**Excitatory-Inhibitory Balance within EEG Microstates and Resting-state fMRI Networks: Assessed via Simultaneous PET-MR-EEG Imaging**

**Rajkumar R^1,2,3^, Régio Brambilla C^1,2,3*^,** **Veselinović T^2*^, Bierbrier J^1,4^, Wyss C^1,5^,**

**Ramkiran S^1^, Orth L^1,2^, Lang M^6^, Rota Kops E^1^, Mauler J^1^, Scheins J^1^, Neumaier B^6^, Ermert J^6^, Herzog H^1^, Langen KJ^1,3,7^, Binkofski F^8^, Lerche C^1^, Shah NJ^1,3,8,9^, Neuner I^1,2,3*^**

^1^Institute of Neuroscience and Medicine 4, INM-4, Forschungszentrum Jülich, 52425 Jülich , Germany

^2^Department of Psychiatry, Psychotherapy and Psychosomatics, RWTH Aachen University, 52074 Aachen,Germany

^3^JARA-BRAIN, 52074 Aachen, Germany.

^4^Department of Electrical and Computer Engineering, McMaster University, Hamilton,

ON L8S 4L8, Ontario, Canada.

^5^Department for Psychiatry, Psychotherapy and Psychosomatics Social Psychiatry, University Hospital of Psychiatry Zurich, 8008 Zurich, Switzerland

^6^Institute of Neuroscience and Medicine 5, INM-5, Forschungszentrum Jülich, 52425 Jülich Germany

^7^Department of Nuclear Medicine, RWTH Aachen University, 52074 Aachen, Germany

^8^Department of Neurology, RWTH Aachen University, 52074 Aachen, Germany

^9^Institute of Neuroscience and Medicine 11, INM-11, Forschungszentrum Jülich, 52425 Jülich, Germany

*Corresponding author: Prof. Dr. Irene Neuner (*[*i.neuner@fz-juelich.de*](mailto:i.neuner@fz-juelich.de)*) (Tel.: +49 2461 61-6356, Fax: +49 2461 61-1919)*

**Equal contribution*

Key words: mGluR5, GABA_A_, DMN, microstates, trimodal, PET-MR-EEG, resting state

# Supplementary Figures


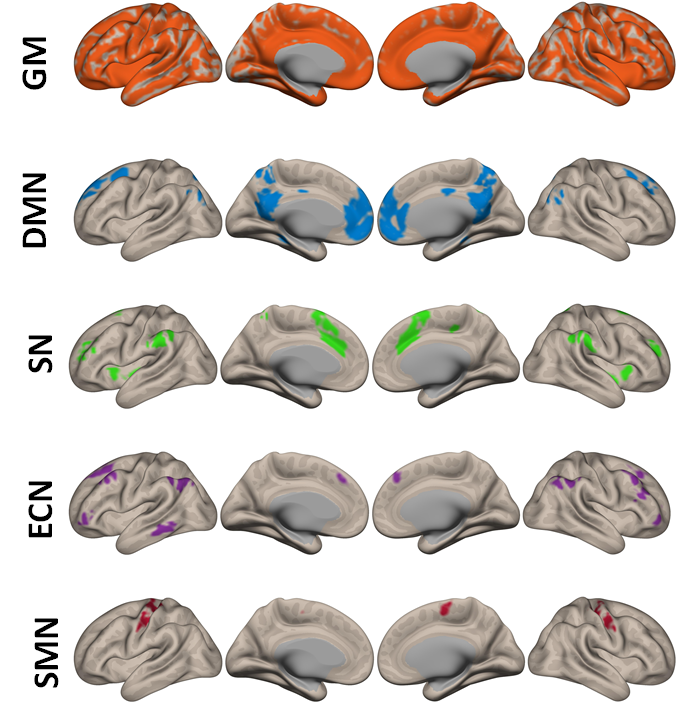


Supplementary Fig. 1: Masks of whole brain grey matter (GM) and resting state networks (default mode network (DMN), salience network (SN), executive control network (ECN) and sensory motor network (SMN) ) obtained from an atlas of 90 functional regions of interest (fROIs) (Shirer *et al.*, 2012).


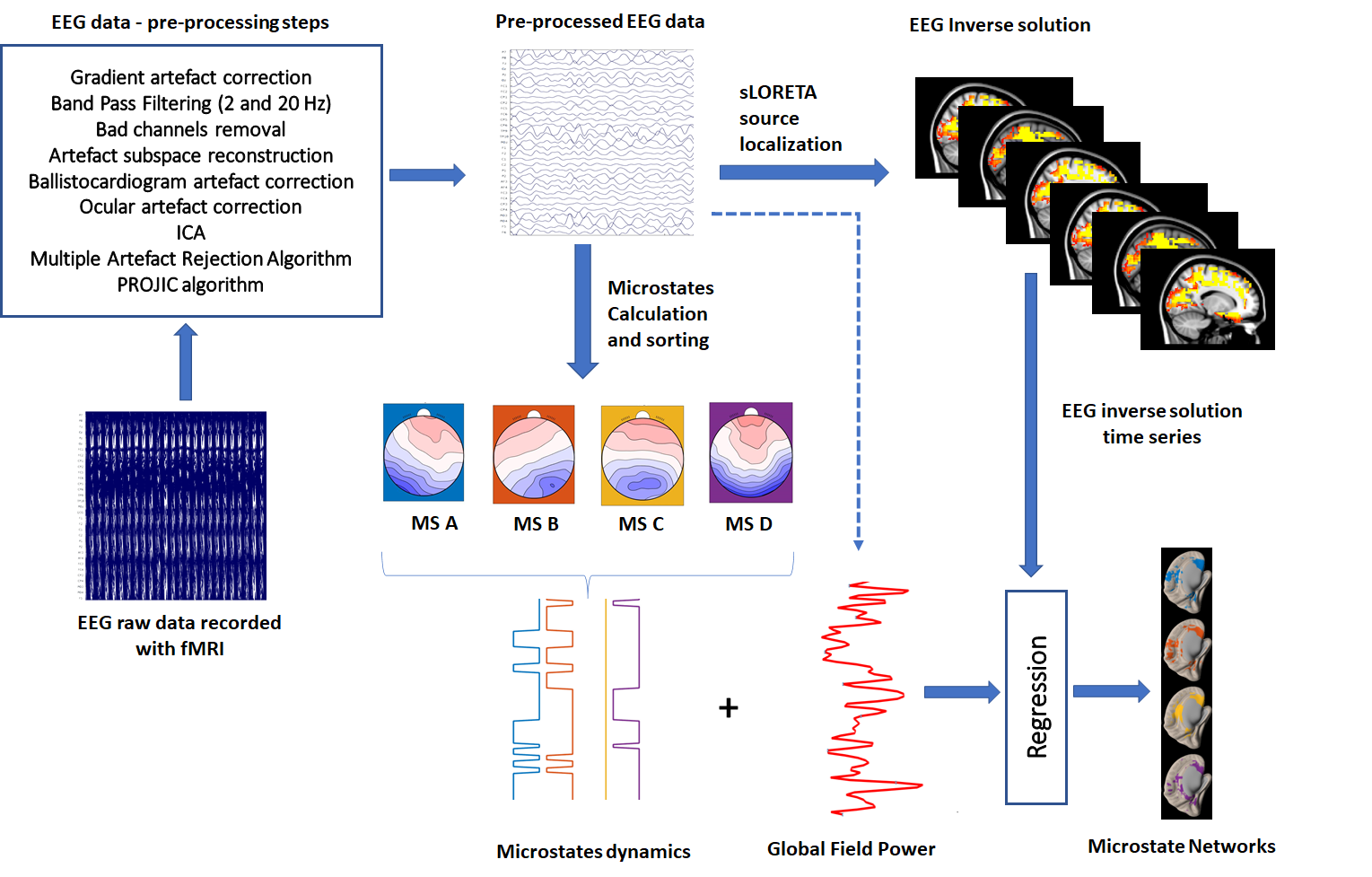


Supplementary Fig. 2: Preprocessing steps and TESS method (Custo et al., 2014) to identify microstate networks. The resting state EEG data recorded along with fMRI is subjected to preprocessing steps to remove artefacts. The microstates were identified and sorted from artefact free EEG data. Concurrently, the inverse solution was computed for artefact corrected EEG data using sLORETA technique (Pascual-Marqui, 2002). The microstate networks were identified via general linear modelling (GLM) of EEG inverse solution time series with microstate dynamics and global field power of EEG signals.

# Supplementary Tables

Supplementary Table 1. Pearson Correlation r-value between averaged PET and microstates.

|  | **mGLUR5** | **GABA_A_** | **FDG-SUV** |
| --- | --- | --- | --- |
| **Microstate A** | 0.16** | 0.27** | -0.03** |
| **Microstate B** | 0.13** | 0.24** | -0.1** |
| **Microstate C** | 0.06** | 0.23** | -0.05** |
| **Microstate D** | 0.15** | 0.27** | -0.08** |
|  |  |  |  |

*p < 0.01

Supplementary Table 2. Pearson Correlation r-value between averaged PET and fMRI measures in the GM and core RSNs regions.

|  | | **fMRI DC** | | **fMRI ReHo** | **fMRI fALFF** |
| --- | --- | --- | --- | --- | --- |
| **FDG SUV in GM** | | 0.49* | | 0.57* | 0.42* |
| **GABA_A_ in GM** | | 0.51* | | 0.59* | 0.43* |
| **mGLUR5 in GM** | | 0.36* | | 0.38* | 0.41* |
| **FDG SUV in DMN** | | 0.23* | | 0.52* | 0.26* |
| **GABA_A_ in DMN** | | 0.43* | | 0.62* | 0.44* |
| **mGLUR5 in DMN** | | 0.10* | | 0.32* | 0.30* |
| **FDG SUV in SN** | | 0.27* | | 0.28* | 0.24* |
| **GABA_A_ in SN** | | 0.29* | | 0.31* | 0.21* |
| **mGLUR5 in SN** | | 0.21* | | 0.40* | 0.17* |
| **FDG SUV in ECN** | | 0.43* | | 0.41* | 0.35* |
| **GABA_A_ in ECN** | | 0.64* | | 0.66* | 0.65* |
| **mGLUR5 in ECN** | | 0.42* | | 0.53* | 0.52* |
|  |  | |  | |  |

*p < 0.01
