# Supplementary material for: Excitatory–inhibitory balance within EEG microstates and resting-state fMRI networks: assessed via simultaneous trimodal PET–MR–EEG imaging
Source: Transl Psychiatry. 2021 Jan 18;11:60. doi: 10.1038/s41398-020-01160-2 (PMC7813876; doi:10.1038/s41398-020-01160-2)
Supplement: Supplementary file 2 — Supplementary Table 2 [file 41398_2020_1160_MOESM2_ESM.docx]

Supplementary Table 2. Pearson Correlation r-value between averaged PET and fMRI measures in the GM and core RSNs regions.

|  | | **fMRI DC** | | **fMRI ReHo** | **fMRI fALFF** |
| --- | --- | --- | --- | --- | --- |
| **FDG SUV in GM** | | 0.49* | | 0.57* | 0.42* |
| **GABA_A_ in GM** | | 0.51* | | 0.59* | 0.43* |
| **mGLUR5 in GM** | | 0.36* | | 0.38* | 0.41* |
| **FDG SUV in DMN** | | 0.23* | | 0.52* | 0.26* |
| **GABA_A_ in DMN** | | 0.43* | | 0.62* | 0.44* |
| **mGLUR5 in DMN** | | 0.10* | | 0.32* | 0.30* |
| **FDG SUV in SN** | | 0.27* | | 0.28* | 0.24* |
| **GABA_A_ in SN** | | 0.29* | | 0.31* | 0.21* |
| **mGLUR5 in SN** | | 0.21* | | 0.40* | 0.17* |
| **FDG SUV in ECN** | | 0.43* | | 0.41* | 0.35* |
| **GABA_A_ in ECN** | | 0.64* | | 0.66* | 0.65* |
| **mGLUR5 in ECN** | | 0.42* | | 0.53* | 0.52* |
|  |  | |  | |  |

*p < 0.01
