# Supplementary figures and images for: Excitatory–inhibitory balance within EEG microstates and resting-state fMRI networks: assessed via simultaneous trimodal PET–MR–EEG imaging
Source: Transl Psychiatry. 2021 Jan 18;11:60. doi: 10.1038/s41398-020-01160-2 (PMC7813876; doi:10.1038/s41398-020-01160-2)

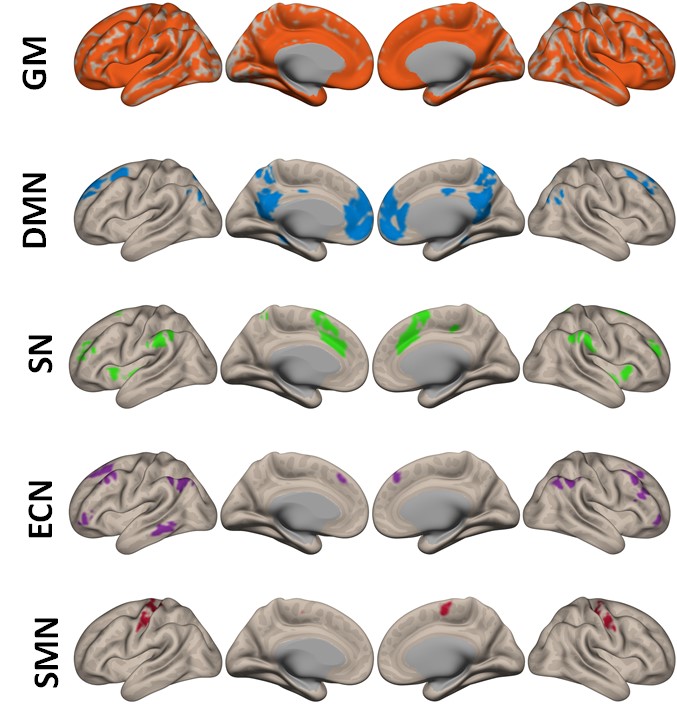

Supplement: Supplementary file 3 — Supplementary Fig. 1 [file 41398_2020_1160_MOESM3_ESM.jpg]

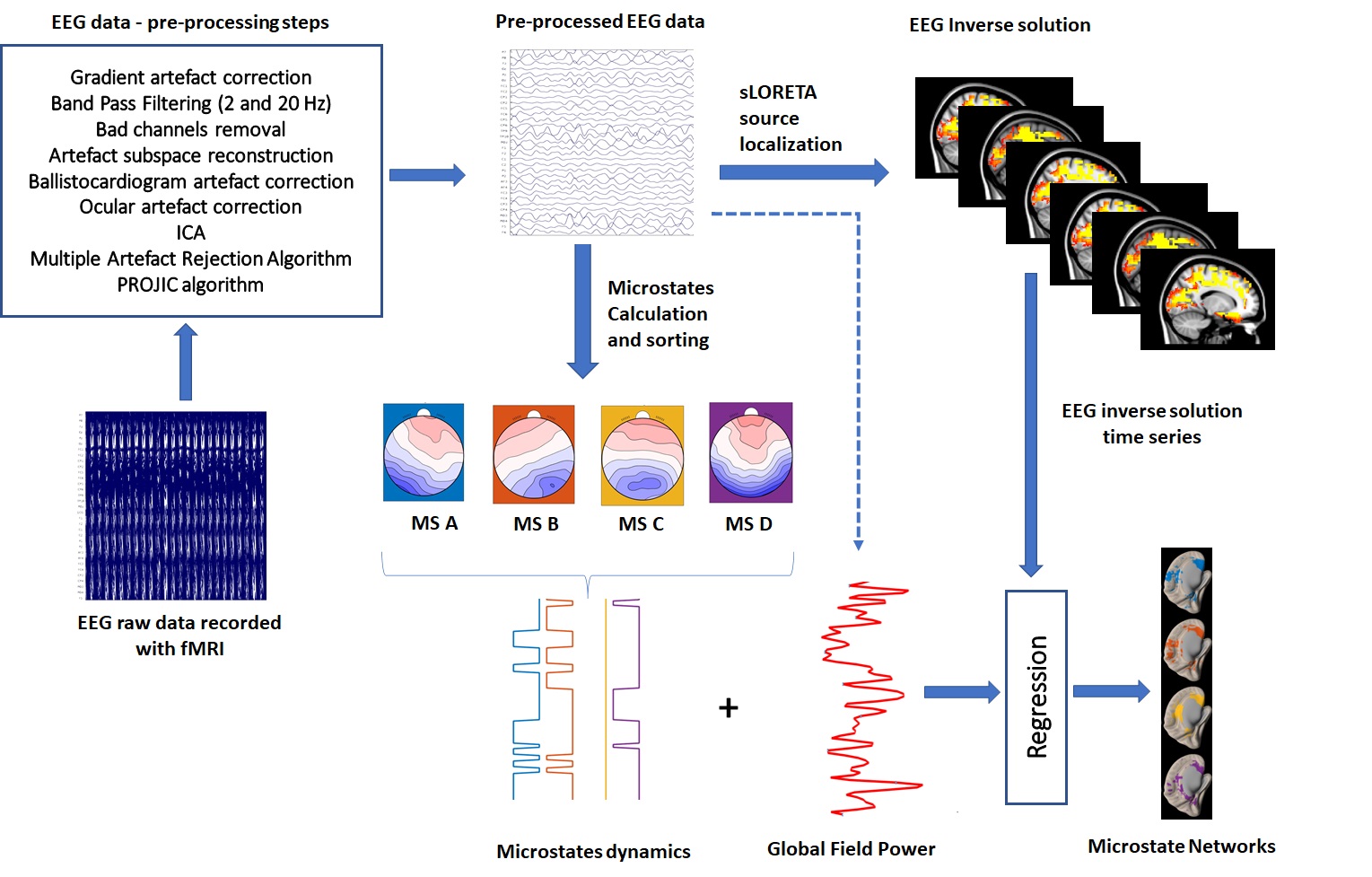

Supplement: Supplementary file 4 — Supplementary Fig. 2 [file 41398_2020_1160_MOESM4_ESM.jpg]
